# Ecological Insights to Track Cytotoxic Compounds among *Maytenus ilicifolia* Living Individuals and Clones of an Ex Situ Collection

**DOI:** 10.3390/molecules24061160

**Published:** 2019-03-23

**Authors:** Daniel Petinatti Pavarini, Denise Medeiros Selegato, Ian Castro-Gamboa, Luiz Vitor Silva do Sacramento, Maysa Furlan

**Affiliations:** 1 Instituto de Química, University Estadual Paulista-UNESP, Rua Prof. Francisco Degni, 55, Quitandinha, Araraquara, SP 14800-060, Brazil; denisemselegato@gmail.com (D.M.S.); ian.castro@gmail.com (I.C.-G.); 2 Institute for Global Food Security, School of Biological Sciences, Queen’s University Belfast, Cloreen Park, Malone Road, Belfast BT9 5HN, UK; 3 Faculdade de Ciências Farmacêuticas, University, Estadual Paulista-UNESP, Rod. Araraquara-Jaú km 1, Araraquara, SP 14801-903, Brazil; lvss@fcfar.unesp.br

**Keywords:** quinonemethide triterpenes, sesquiterpene pyridine alkaloids, chemical diversity, Celastraceae, Hierarchical Clustering Analysis (HCA)

## Abstract

Biodiversity is key for maintenance of life and source of richness. Nevertheless, concepts such as phenotype expression are also pivotal to understand how chemical diversity varies in a living organism. Sesquiterpene pyridine alkaloids (SPAs) and quinonemethide triterpenes (QMTs) accumulate in root bark of Celastraceae plants. However, despite their known bioactive traits, there is still a lack of evidence regarding their ecological functions. Our present contribution combines analytical tools to study clones and individuals of *Maytenus ilicifolia* (Celastraceae) kept alive in an ex situ collection and determine whether or not these two major biosynthetic pathways could be switched on simultaneously. The relative concentration of the QMTs maytenin (**1**) and pristimerin (**2**), and the SPA aquifoliunin E1 (**3**) were tracked in raw extracts by HPLC-DAD and ^1^H-NMR. Hierarchical Clustering Analysis (HCA) was used to group individuals according their ability to accumulate these metabolites. Semi-quantitative analysis showed an extensive occurrence of QMT in most individuals, whereas SPA was only detected in minor abundance in five samples. Contrary to QMTs, SPAs did not accumulate extensively, contradicting the hypothesis of two different biosynthetic pathways operating simultaneously. Moreover, the production of QMT varied significantly among samples of the same ex situ collection, suggesting that the terpene contents in root bark extracts were not dependent on abiotic effects. HCA results showed that QMT occurrence was high regardless of the plant age. This data disproves the hypothesis that QMT biosynthesis was age-dependent. Furthermore, clustering analysis did not group clones nor same-age samples together, which might reinforce the hypothesis over gene regulation of the biosynthesis pathways. Indeed, plants from the ex situ collection produced bioactive compounds in a singular manner, which postulates that rhizosphere environment could offer ecological triggers for phenotypical plasticity.

## 1. Introduction

It has been established that biodiversity is a central theme in several political and scientific forums. Over the last decades, the impact that biodiversity has on life maintenance on the planet has furnished several theories, such as the Gaia theory, proposed by Lovelock in the 60s, and the Anthropocene geological era theory, proposed by Crutzen [1,2]. 

Biodiversity reached the headlines of academic topics building on the principle that it provides richness. In Nature, this intrinsic value of biodiversity extends beyond ecosystem variability, ecological services and genetic heritage and determines both individual viability and community plasticity. For instance, Brazil is a megadiverse country that hosts 10–20% of all known living species in the world. Over the years, the evaluation of biodiversity [3,4,5,6] has culminated in bio-economic development [7] and fostered Brazilian public policies towards conservation of this resource [5]. Recent findings on the Brazilian “Mata Atlântica” rainforest, alongside the efforts of the FAPESP Research Program on Biodiversity Characterization, Conservation, Restoration and Sustainable Use (BIOTA, http://www.biota.org.br/en/) are a handful of successful study cases on how biodiversity data could be used for conservation and sustainable use of Brazilian natural resources. For example, our research group has recently created the NuBBEDB [8,9], the first natural product database of Brazilian biodiversity. This library, which is nowadays a repository of Chemspider (http://www.chemspider.com/DatasourceDetails.aspx?id=806), covers validated multidisciplinary information, geographical locations, species sources, chemical descriptors, spectroscopic NMR data and pharmacological properties of over 2,000 natural compounds and derivatives, contributing to enhance both dereplication and drug discovery studies [8,9].

Despite this self-standing paradigm of biodiversity as a provider, side concepts are also pivotal to understanding how chemical diversity is expressed in living organisms. In Nature, lifeforms display morphological and molecular traits that exhibit both qualitative and quantitative differences. These variations are often related to the ecosystem in which these organisms are embedded, encoding genes that are expressed differently according to the biotic and abiotic conditions of the surroundings [10]. 

Among the ecosystem features, the habitat plays a key role in driving the physiology of wild type plant species [11]. Hence, it has been established that biosynthesis pathways can be triggered by specific ecological interactions [12], resulting in distinct metabolic profiles in different individuals of the same plant species. Examples of this plasticity have been extensively reported for domestic plants, such as wine grapes (*Vitis vinifera*: Vitaceae [13]) and apples (*Malus domestica*: Rosaceae [14]). However, only a few studies on literature address wild plants endemic to the neotropics, such as reported for *Casearia sylvestris* (Salicaceae) [15].

Specifically, for one particular organism, time-dependent events can also cause a huge diversity of effects inducing plasticity and bioactive compound production [16]. Ontogenesis is a central one. For example, it has been reported that younger species of flowering plant might display a unique biosynthetic pathway that operates exclusively during a particular age [17,18]. 

In the Celastraceae, the production of terpenes shifts among different plant species, tissues and season, as reviewed by Alvarenga and Ferro [19]. For instance, our group has shown that the biosynthesis of the triterpene friedelin happens in the leaves but produces the precursors of quinone methide triterpenes, which are synthesized in the roots [20]. These variations raise the hypothesis of time-dependent and age-related chemical behaviour, which have been widely reported in the current literature [16] but has not been proved yet within this family. 

Most commonly found bioactive metabolites in the root bark extracts of Celastraceae species are quinonemethide triterpenes (QMTs) and sesquiterpene pyridine alkaloids (SPAs). Quinonemethide triterpenes display cytotoxic, anti-ulcerogenic and antioxidant effects, as well as anticancer activity against different human cancer cell lines [21,22,23,24,25,26,27]. Similarly, the sesquiterpene pyridine alkaloids (SPAs) display insect antifeedant [28], insecticidal [29], cytotoxic [30], immunosuppressive [26], anti-HIV [26], antiprotozoal [31], anti-hepatitis C [32] and antitumor [33] activities.

**Figure 1:**
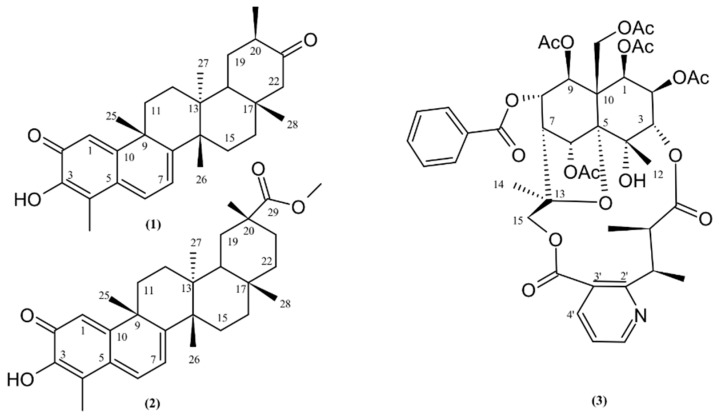
Structures of QMTs maytenin (**1**) and pristimerin (**2**) and the SPA aquifoliunin E1 (**3**) from *Maytenus ilicifolia* with carbons numbered accordingly.

In this topic, we aim to expose the relevance of multi-trophic interactions and ontogenesis in determining the richness of bioactive classes of compounds among ex situ collections of *Maytenus ilicifolia* Mart. ex Reissek (Celastraceae). This specie is generally known in South America as “Espinheira-santa” and is the most used *Maytenus* species in traditional medicine, where its leaves infusion exhibits high antiulcerogenic effects [34]. The present phytochemistry study aimed to determine whether QMT and SPA levels are different among clones and individuals of *M. ilicifolia* kept alive in an ex situ collection. The relative concentration of the QMTs maytenin (**1**) and pristimerin (**2**), and the SPA aquifoliunin E1 (**3**) [Figure 1] were tracked in raw extracts by HPLC-DAD and ^1^H-NMR. Then, Hierarchical Clustering Analysis (HCA) was used to gather groups of individuals according their ability to accumulate these compounds. Summarily, this communication relies on straightforward analytical tools to determine how different can the secondary metabolites profile patterns be within common environmental conditions acting upon individuals.

## 2. Results and Discussion

### 2.1. Measurement of QMTs and SPA in Root Barks from M. ilicifolia

The first analytical analysis was made by loading raw extracts onto TLC plates. Results are displayed in the Supplementary Material. The snapshots of all TLCs run show the chemical diversity among the plants from the ex situ collection. Particularly, the highest diversity of all, measured by the numbers of different spots, was displayed by adult with shooting (PAVARINI I-III). 

Among these qualitative shifts, it can be seen that all spots present in the eleventh line from left to right, corresponding to a fraction enriched with maytenin (**1**, PubChemCID 86280086) and pristimerin (**2**, PubChemCID 101520), are conserved through all plants. A purple tone on the spots was suggestive of terpene occurrence [29].

**Figure 2:**
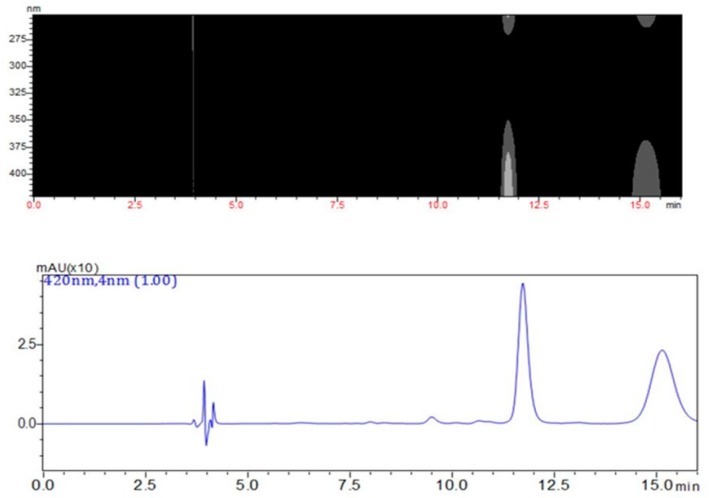
Above, diode array detection of the chromatogram acquired within the wavelength detection window. The extract of PAVARINI I afforded this result. Maximum λ values can be noted at 270 nm and 420 nm. Below, extracted chromatogram at λ 420 nm displaying two QMTs, maytenin (Rt 11.7 min) and pristimerin (Rt = 15.0 min), and one SPA, aquifoliunin E1 (Rt 9.6 min).

Regarding the results of the HPLC-DAD method, it can be said that neither pristimerin or maytenin were found in the majority of individuals, as depicted in Table S2 (available in the Supplementary Material). The extracted chromatogram at 420 nm exhibited peaks for the SPA aquifoliunin E1 (**3**, Rt 9.6 min), maytenin (**1**, Rt 11.7 min) and pristimerin (**2**, Rt 15.0 min). The DAD monitoring showed that the peaks had a λ_max_ at 420 nm (Figure 2). The major part of individuals also displayed contents of both QMT metabolites.

In many domestic plants, the enzymatic activity of CYP450 during the biosynthesis of QMT yields reactions on the E ring, mostly at positions 19, 20, 21 and 22 [35]. There is a suggestive involvement of this enzymes in the functionalization of QMT, however it remains an unresolved matter in biosynthetic pathway studies.

Results from the ^1^H-NMR spectra of all samples showed similar chemical shifts between maytenin (**1**) and pristimerin (**2**), with major differences on the aromatic signals of rings A and B, the presence of a singlet from the methoxyl hydrogens of pristimerin at δ3.48 (*s*, 3H, H-29) and the significant shift in the methyl group at H-27. NMR spectra of *M. ilicifolia* raw extract (CDCl_3_, 600 MHz) showed five maytenin (**1**) signals at δ 6.28 (*d*, *J* 7.2 Hz, 1H, H-7), δ 6.46 (*d*, *J* 0.8 Hz, 1H, H-1), δ 6.97 (*dd*, *J* 7.2 and 0.8 Hz, 1H, H-6), δ 1.38 (*s*, 3H, H-25) and δ 2,14 (*s*, 3H, C-23); and six pristimerin (**2**) signals at δ 6.31 (*d*, *J* 7.1 Hz, 1H, H-7), δ 6.48 (*d*, *J* 0.8 Hz, 1H, H-1), δ 6.95 (*dd*, *J* 7.1 and 0.8 Hz, 1H, H-6), δ 2.16 (*s*, 3H, C-23), δ 1.36 (*s*, 3H, H-25) and δ 0.47 (*s*, 3H, H-27), with major peaks highly convoluted between δ 0–2.5. Confirmatory analysis by Heteronuclear Multiple Bond Correlation (^1^H-^13^CHMBC) and Heteronuclear Single Quantum Coherence (^1^H-^13^CHSQC) have also been assessed for both metabolites and are displayed in Table S2 (Supplementary Materials). Data from literature, including data generated by our own research group, was used for comparison [36,37].

**Figure 3:**
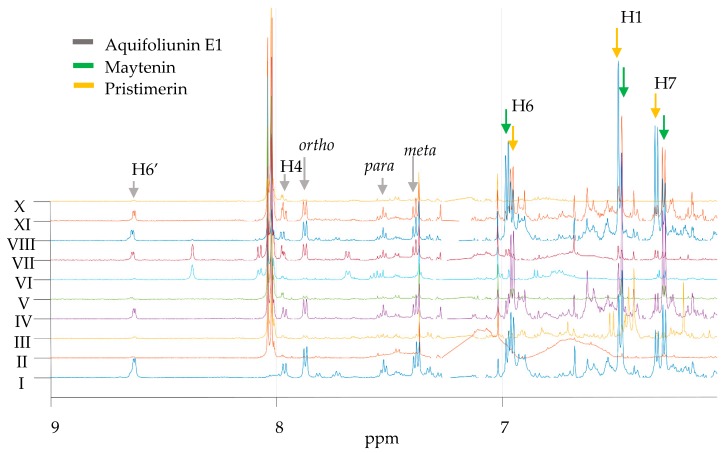
^1^H-NMR spectra of PAVARINI I-X raw extracts (hexane–ethyl acetate 8:2) and the detection of SPA aquifoliunin E1 signals at δ 8.62 (*dd*, *J* 4.7 Hz, 1.8, 1H, H-6′), δ 7.96 (*dd*, *J* 7.8; 1.8 Hz, 1H, H-4′), δ 7.87 ppm (*dd*, *J* 8.0 and 1.0 Hz, 2H, *ortho*), δ 7.52 ppm (*t*, *J* 8.0 Hz, 1H, *para*) and δ 7.38 ppm (*dd*, *J* 7.4 and 8.2, 2H, *meta*); maytenin signals at δ 6.97 (*dd*, *J* 7.2 and 0.8 Hz, 1H, H-6), δ 6.46 (*d*, *J* 0.8 Hz, 1H, H-1) and δ 6.28 (*d*, *J* 7.2 Hz, 1H, H-7); and pristimerin signals at δ 6.95 (*dd*, *J* 7.1 and 0.8 Hz, 1H, H-6), δ 6.48 (*d*, *J* 0.8 Hz, 1H, H-1) and δ 6.31 (*d*, J 7.1 Hz, 1H, H-7).

The search for SPA proton signals were based in two major spin systems from the spectra aromatic region: (1) pyridinic nucleus at δ 7.96 (dd, J 7.8 and 1.8 Hz, 1H, H-4′), δ 7.18 (dd, J 7.8 and 4.7 Hz, 1H, H-5′) and δ 8.62 (dd, J 4.7 Hz and 1.8, 1H, H-6′); and (2) benzoate nucleus at δ 7.87 (dd, J 8.0 and 1.0 Hz, 2H, ortho), δ 7.52 (t, J 8.0 Hz, 1H, para) and δ 7.38 (dd, J 7.4 and 8.2, 2H, meta). The SPA signals were not easily detected in the raw extract ^1^H-NMR spectra of samples of M. ilicifolia. Detectable SPA signals were identified only in PAVARINI I, IV, VII, VIII and IX, with major production of SPA on the adult plant PAVARINI I (Figure 3). Confirmatory ^1^H-^13^CHSQC, ^1^H-^13^CHMBC and 1DTOCSY, as well as the complete set of chemical shifts of SPA are displayed in the Supplementary Material as Table S4.

### 2.2. Hierarchical Clustering Analysis (HCA)

**Figure 4:**
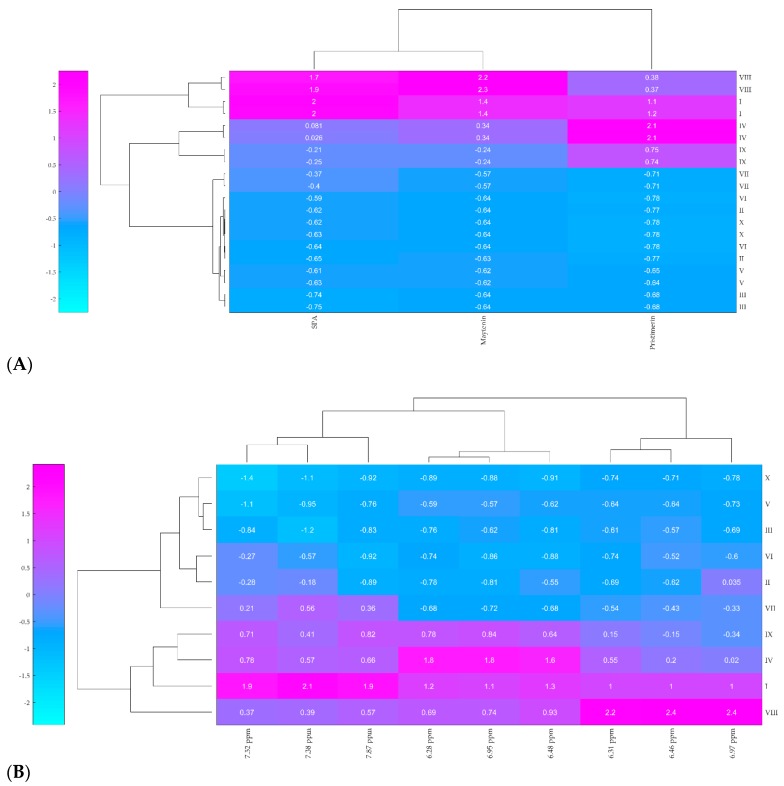
HCA of chemical data. Samples with similar color (hence, similar Euclidian distances) are clustered together. (**A**) Clustergram from HPLC-DAD data. Input was the total integrated areas of SPA, pristimerin and maytenin peaks displayed in each chromatogram. (**B**) Clustergram from ^1^H-NMR data. Input was the total integrated areas of SPA, pristimerin and maytenin aromatic signals displayed in each spectrum: SPA aquifoliunin E1 signals [δ 7.87 (*dd*, *J* 8.0 and 1.0 Hz, 2H, *ortho*), δ 7.52 (*t*, *J* 8.0 Hz, 1H, *para*) and δ 7.38 (*dd*, *J* 7.4 and 8.2 Hz, 2H, *meta*)]; pristimerin signals [δ 6.95 (*dd*, *J* 7.1 and 0.8 Hz, 1H, H-6), δ 6.48 (*d*, *J* 0.8 Hz, 1H, H-1) and δ 6.31 (*d*, *J* 7.1 Hz, 1H, H-7)] and maytenin signals [δ 6.97 (*dd*, *J* 7.2 and 0.8 Hz, 1H, H-6), δ 6.46 (*d*, *J* 0.8 Hz, 1H, H-1)] and δ 6.28 (*d*, *J* 7.2 Hz, 1H, H-7).

The HCA results are displayed as a dendrogram combined with the ordered heatmap that represents the Euclidean distance for each pair of samples (Figure 4). Samples clustered together display a high similarity in the production of QMT and SPA, whereas distant samples show low correlation. The heatmap was plotted according to the relative abundance of the bioactive metabolites, gradually varying their concentration from high (pink) to low (light blue).

The HCA analysis grouped three main clusters based on the production of QMT and SPA. This result was corroborated both by HPLC-DAD and ^1^H-NMR data (Figure 4A,B, respectively). The first cluster corresponds to the samples VIII and I that produced a high concentration of maytenin and aquifoliunin E1 (HPLC-DAD maytenin total integrated area of 4,064,893.00 to 5,750,392.00) and a moderate to high concentration of pristimerin (HPLC-DAD pristimerin total integrated area of 3,495,843.00 to 5,856,003.00), including the sample PAVARINI VIII, which produces the biggest amount of maytenin and aquifoliunin E1 among all samples.

The second cluster, formed by PAVARINI IV and IX, represent samples that produce a low to moderate concentration of maytenin and aquifoliunin E1 (HPLC-DAD maytenin total integrated area of 810,955.00 to 1,956,681.00) and a high concentration of pristimerin (HPLC-DAD pristimerin total integrated area of 4,600,934.00 to 8,556,043.00) and includes the sample IV that produces the biggest amount of pristimerin.

The last cluster, placed on the higher dendogram edge, opposite to the first two cluster, includes samples II, III, V, VI, VII and X and represents samples that produce low concentration of all metabolites, including maytenin (total integrated area of 13,085.00 to 27,533.00) and pristimerin (21,480.00 to 433,813.00). The biggest difference in this cluster was the production of aquifoliunin E1, in which the concentration varies between absent (samples III, V and X) and moderate (II, VI and VII).

These results show that maytenin and pristimerin production is not dependent on the plants’ age, as observed in the clustering patterns. These novel data, herein firstly presented, disprove the hypothesis that QMT biosynthesis is dependent to the plant’s age. Hence, ontogenetic shifts in biosynthesis enzymes, such as CYP450, are unlikely to play roles in the phenotypical plasticity of *M. ilicifolia*. Furthermore, the clustering was also not dependent on the reproduction of the plant (clones/individuals). This finding, in particular, might reinforce the hypothesis over gene regulation of the biosynthesis pathways. Currently the plant science literature reports that triterpene production on *Bupleurum falcatum* L. (Apiaceae) can be epigenetically altered, as β-amirin synthase production can be overstimulated via jasmonate treatment [38].

### 2.3. Discussion

The harvesting of plant material made it possible to assemble two set of living organisms: (a) a genetic diverse pool of sexually reproduced plants and (b) a clonal pool of individuals reproduced by shootings. Hence, our comparative analysis of single individuals allowed us to access plants with different phenotype, different genotype and identical genotype. The extracts display quite different chemical traits from each other. As expected, the presence/absence of differences in QMT and SPA levels were observed among the population. Clonal and individual analysis of plants form ex situ collection from “BG-PSS” show an extensive occurrence of QMTs. This might indicate that only quantities are shiftable according to the age of the plants. In such case, the phenotypical plasticity might be involved in individual responses. Furthermore, the extensive presence of QMTs and low abundance of SPAs suggests a constitutive accumulation of QMTs, similar to the accumulation of sapogenins in *Medicago truncatula* roots [39]. It has been already reported that *Maytenus rigida* root bark extracts display antimicrobial saponins [40].

The plants coded PAVARINI I, IV, VIII and IX were the ones with higher amounts of QMTs. Similarly, those samples, along with PAVARINI VII, also displayed low production of SPA. Samples I and VIII displayed the higher concentration of both QMTs and SPAs.

Even though the extracts were made targeting the QMT extraction, our dereplication experiments also detected the pyridinic and benzene rings of the alkaloids in several samples. So far, this result is the first in the current literature to report the occurrence of two bioactive classes of compounds in both adult and young *Maytenus ilicifolia*.

The results displaying similar secondary metabolism profiles for clones and individuals support the hypothesis of gene regulation of the biosynthesis pathways. Furthermore, the differences in the levels of maytenin (**1**) and pristimerin (**2**) might also be related to the semiochemical aspects of these bioactive compounds in the rhizosphere. Commonly, the isolated compounds from *Maytenus* spp. root bark extracts had their bioactivity tested in drug discovery models. However, the role that QMTs might play in ecological interactions might be hypothesized due to the highly toxic trait of quinone functions [41] or to the known function of triterpenes as plant defenses [18]. Recent findings achieved in collaboration with our research group proved that an effective antimicrobial activity can be observed for maytenin and 22-β-hydroxymaytenin isolated from the Celastraceae species *Peritassa laevigata* [34].

## 3. Experimental

### 3.1. Sampling

Individuals were monitored since their transplant from the original habitat, during the late 90s up to nowadays. Hence, sexual reproduction, through bird’s dispersion of seeds, and asexual reproduction from branches and root shootings, were determined through simple observation of each piece of the collection. All the plants of the collection maintained in the botanical garden were sampled in the present study. Height description and morphological traits of each individual are available as Table S1 and Figure S2 in the Supplementary Material.

Side shootings of roots were harvested, and their bark was separated from the inner parts of the root. For this harvest, the root was unburied, the bark separated and collected, and the remaining parts buried again. There was no significant injury to the plants in the collection, which are still kept alive. After sampling, the barks were rinsed under water flow to remove soil particles and allowed to dry at controlled temperature of 36 °C for 48 h.

Samples were harvested in an ex situ collection that comprises ten different specimens of *Maytenus ilicifolia*, as described in Table 1. A total of 10 individuals kept alive in ex situ conditions at the botanical garden of Pharmaceutical Science School/UNESP (BG-PSS) (GPS S: -21.814885, W: -48.201091) were codified (Table 1).

According the Resolution 29 from 2007, all harvest procedures are exempt of prior permission from the appropriate deprtment of the Brazilian Environment Ministry, the MMA–ICMbio. However, MMA–ICMbio has granted us an allowance for all field activities (49727-2). One botanical voucher was included in the in-house collection under the code DPP005 and is available for consultation at the Institute of Chemistry from the São Paulo State University-UNESP, Araraquara-SP, Brazil. Furthermore, another voucher was deposited under number HRCB 68663 at the “Herbário Rioclarense” in the São Paulo State University (UNESP), Rio Claro, SP, Brazil.

### 3.2. Metabolite Extraction

The metabolite extraction was done after grinding the dried material. We have chosen a mixture of hexane-ethyl acetate (8:2 *v*/*v*) as the extraction solvent in order to target lipophilic compounds. Extraction was repeated three times and the total volume of the extractor solvent used was equivalent to six times the mass of powdered plant material. The dry material yielded 1.0–8.0 g. The resulting organic extract was allowed to dry overnight. A viscous and oily matter colored yellowish-orange (1.00–150.00 mg of plant extract) was furnished after drying. Particles at the bottom and orange crystals at the top were displayed. The amount yielded is recorded at Table S1 in the Supplementary Material.

### 3.3. General Analytical Procedures

Thin layer chromatography (TLC) experiments were conducted using hexane-ethyl acetate (8:2 *v*/*v*) as mobile phase. Detection was achieved using UV light and further *p*-anisaldehyde spraying followed by heating at 100°. For High Performance Liquid Chromatography–Diode Array Detector (HPLC-DAD) analysis, an LC-10 AD instrument (Shimadzu Europa GmbH, Duisburg, Germany) equipped with a SPD-M 10 AVP detector and a Gemini C_18_, column [Phenomenex^®^, Torrance, CA, USA), 5 μm (250 × 4.6 mm)] were used. The mobile phase was methanol-water-phosphoric acid (80:20:1 *v*/*v*/*v*), used in isocratic mode (flow at 0.8 mL min^−1^). Total run time was 16 min and the detection window was UV-Vis >254 nm and <420 nm. Column oven was kept at 35 °C. Prior to injection on the column, the extracts went through a solid phase extraction (SPE) procedure using Chromabond^®^, from ThermoFisher (Waltham, MA, USA), C_18_ cartridges. The SPE cartridges were conditioned using EtOH-H_2_O (85:15 *v*/*v*). Afterward, 10 mg of the dried extracts after solvent removal were loaded. At the end of clean-up treatment, 200 μL of the yield solution was dissolved in 1.300 μL of HPLC mobile phase.

NMR spectra were acquired using an Advance DBO Probe (Bruker, Billerica, MA, USA) under an 11.6 T magnetic field. Samples were prepared solubilizing 10.00 ± 0.1 mg from each sample in 700 µL of deuterated chloroform-d (CDCl_3_, Cambridge Isotope Laboratories, Tewksbury, MA, USA) using 5 mm inner diameter tube. For ^1^H-NMR acquisition (CDCl_3_), 90°. pulse sequence [zg90 (Bruker, Billerica, MA, USA)] analysis parameters were time domain (TD) 65 k, number of scans (NS) 16, relaxation delay (d1) 1.00 s, spectral width (SW) 20 ppm, dummy scans (DS) 4; and temperature 295.2 K. For HMBC and HSQC acquisition, analysis parameters were, respectively for ^1^H (f2) and ^13^C (f1), spectrometer frequency 600.13 and 150.91 MHz; TD 1024 and 1024; SW 20 and 230 ppm; NS 24; d1 1.5 s and temperature 295.2 K. Pulse sequences used were (i) phase-sensitive ge-2D HMBC using a two-fold low-pass J-filter [hmbcetgpl3nd 2D, (Bruker, Billerica, MA, USA] for ^1^H-^13^C HMBC and (ii) phase-sensitive ge-2D multiplicity edited HSQC using PEP and adiabatic pulses with gradients in back-inept [hsqcedetgpsisp2.4, (Bruker, Billerica, MA, USA)] for ^1^H-^13^C HSQC. The long-range coupling constant used for HMBC was 8.5 Hz.

### 3.4. Hierarchical Clustering Analysis (HCA)

HCA is an agglomerative clustering analysis in which the measurement of distance between pairs creates a hierarchy between samples. In this procedure, most similar points are grouped together, creating a dendogram plot that clusters samples based on their similarity. For HCA experiments, samples I-X were clustered, in triplicate, based on the QMTs and SPA production measured by the semi-quantitative HPLC-DAD and ^1^H-NMR data. For this analysis, the similarity was calculated using Euclidean distance between the samples total integrated area. Ward was chosen as the optimum linkage method due to the fewer susceptibility to outlier effects. All data were statistically analyzed using algorithms created in MATLAB software R2017a^®^ (MathWorks, Natick, MA, USA) at a 95% confidence level.

## 4. Conclusions

The dereplication of bioactive metabolites in *M. ilicifolia* raw extracts was successful due to combination of a few practical tasks: (1) rigorous spectroscopy and chromatographic data survey of compounds previously investigated in taxonomically-related species and (2) the proper use of analytical tools and detection channels to narrow information down to the target group of compounds. One of the major efforts of the natural product community around the globe nowadays is to enable fast identification of targeted compounds in complex mixtures [42]. One useful aspect of this paper is as a proof of concept that these two practical tasks can be adopted for rapid selection of botanical vouchers displaying high contents of active terpenes. This successful case stands alongside others such as using of TOCSY for selection of polyketide producer’s bacterial strains [43]. 

Semi-quantitative analysis of the root barks of *M. ilicifolia* by both HPLC-DAD and ^1^H-NMR showed the presence of QMTs in high abundance, whereas SPAs were barely detected in only five samples. Contrary to QMTs, SPAs did not accumulate in root bark tissues extensively, leading us to conclude that the hypothesis of two different biosynthetic pathways operating simultaneously is unlikely.

Moreover, the production of QMTs varied significantly among the samples in the ex situ collection, hence, we might conclude that the QMT contents in root bark extracts of *M. ilicifolia* were not dependent on abiotic effects. Given plants from the same ex situ collection can be thought to be under the same abiotic conditions, effects such as photoperiod, precipitation, seasonal and circadian cycles influence equally every individual.

Hierarchical clustering analysis (HCA) has been consistently efficient in the analysis of natural and multivariate chemical data [44,45,46]. For *M. ilicifolia,* this unsupervised chemometric analysis grouped the samples that shared a similar metabolic production using both HPLC-DAD and ^1^H-NMR data. Some important advantages of this clustering analysis are the possibility to apply high-dimensional data typical from ‘omics’ studies, the possibility of both quantitative and qualitative evaluation and the detection of pleiotropic effects that might influence the chemical variation.

The HCA dendogram showed an extensive occurrence of QMTs, regardless of the plant age. This novel data disproves the hypothesis that QMT biosynthesis is dependent on the plant age. Hence, ontogenetic shifts on biosynthesis enzymes are unlikely to play roles in phenotypical plasticity in *M. ilicifolia*. Furthermore, clustering analysis did not group clones nor same-age samples together, which might reinforce the hypothesis of gene regulation of the biosynthesis pathways. Indeed, our findings indicate that the transcription of the genes encoding the enzymes of these biosynthetic pathways are on in some individuals and off in others.

The clustering of same clones occurred in a relatively high Euclidean distance, hence, low similarity of the QMT and SPA production. Such a distance suggests that genetics might not be driving to this phenotype either. In fact, a Mendelian inheritance might be ruled out.

The recent increase on drug repositioning [47] within the pharmaceutical market should be emphasized here. Since *M. ilicifolia* metabolites display a wide arrange of bioactivities against specific targets, the interest on QMTs should not remain focused on only one disease. The well-known ability of natural products to produce Michael adducts with cysteine residues present in many therapeutic targets, under alkaline pH [48], make QMTs promising repositioning drugs. Rapid evaluation of botanical samples relying on proper use of analytical tools, such as in our present work, is useful to provide materials for such pharmaceutical research.

## Figures and Tables

**Table 1:** *Maytenus ilicifolia* individuals kept alive in ex situ conditions at the botanical garden of Pharmaceutical Science School/UNESP (BG-PSS) (GPS S: -21.814885, W: -48.201091).

Sample	Physiognomy	Type
I	Adult	Individuals
II	Young	Individuals
III	Adult	Clone
IV	Adult	Individuals
V	Young	Individuals
VI	Adult	Clone
VII	Adult	Clone
VIII	Adult	Clone
IX	Adult	Individuals
X	Young	Individuals

## Supplementary Materials

The following are available online.
