# Supplementary material for: Ecological Insights to Track Cytotoxic Compounds among Maytenus ilicifolia Living Individuals and Clones of an Ex Situ Collection
Source: Molecules. 2019 Mar 23;24(6):1160. doi: 10.3390/molecules24061160 (PMC6471723; doi:10.3390/molecules24061160)
Supplement: Supplementary file 1 [file molecules-24-01160-s001.pdf]

Supplementary Material

Ecological Insights to Track Cytotoxic Compounds among *Maytenus Ilicifolia* Living Individuals and Clones of an Ex Situ Collection

Daniel Petinatti Pavarini [1,2,*], Denise Medeiros Selegato [1], Ian Castro-Gamboa [1], Luiz Vitor Silva do Sacramento [3] and Maysa Furlan [1,*]

[1] Instituto de Química, Univ. Estadual Paulista-UNESP, Rua Prof. Francisco Degni, 55, Quitandinha, Araraquara, SP 14800-060, Brazil; Daniel Petinatti Pavarini (D.P.P.); Denise Medeiros Selegato (D.M.S.); Ian Castro-Gamboa (I.C.-G.); Maysa Furlan (M.F.)

[2] Institute for Global Food Security, School of Biological Sciences, Queen's University Belfast, Cloreen Park, Malone Road ,Belfast, BT9 5HN, United Kingdom; Daniel Petinatti Pavarini (D.P.P.)

[3] Faculdade de Ciências Farmacêuticas, Univ. Estadual Paulista-UNESP, Rod. Araraquara-Jaú km 1, Araraquara, SP 14801-903, Brazil; Luiz Vitor Silva do Sacramento

*Correspondence: D.Pavarini@qub.ac.uk or danielpetpav@hotmail.com ((D.P.P.); maysaf@iq.unesp.br (M.F.)

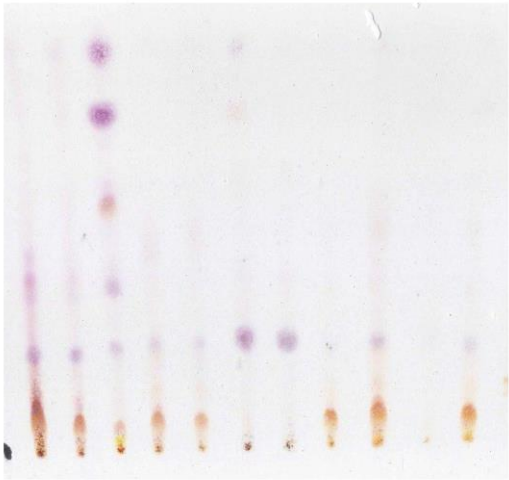

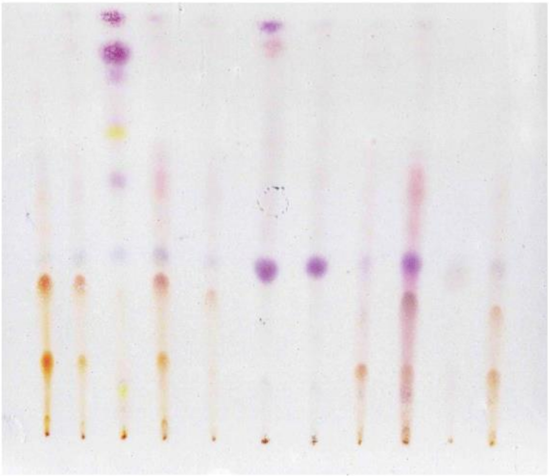

Figure S1. Snapshots of TLCs after spot detection. From left to right the individuals PAVARINI I, II, III, IV, V VI, VII,VIII, IX and X. The eleventh line from left to right is a mixture fraction enriched with pristimerin and maytenin. Mobile phase used was dichloromethane on the left and hexane/ethyl acetate (8:2) on the right.

Table S1. Field records that were registered during harvest at GH-SPS in 2014.

Code	Physiognomy	Dry weight	Extract
"PAVARINI-I"	Adult 300 cm	3.09 g	58 mg
"PAVARINI-II"	Young 80 cm	0.58 g	9 mg
"PAVARINI-III"	Adult with root shooting	1.90 g	50 mg
"PAVARINI-IV"	Adult 200 cm	4.56 g	70 mg
"PAVARINI-V"	Young 30 cm	1.00 g	10 mg
"PAVARINI-VI"	Adult 300 cm	7.42 g	25 mg
"PAVARINI-VII"	Adult 250 cm	2.01 g	8 mg
"PAVARINI-VIII"	Adult 300 cm	5.77 g	150 mg
"PAVARINI-IX"	Adult 200 cm	8.01 g	100 mg
"PAVARINI-X"	Young 15 cm	0.37 g	5 mg

Table S2. Semi-quantitative data from HPLC-DAD analysis. Comparison of levels of QMTs and SPA. Mean value and standard deviation of the total integrated area (10^5).

Code	SPA	Pristimerin	Maytenin
"PAVARINI-I"	1.738 ± 0.018	58.729 ± 0.739	40.483 ± 0.020
"PAVARINI-II"	0.115 ± 0.012	0.753 ± 0.007	0.248 ± 0.021
"PAVARINI-III"	0.043 ± 0.005	3.400 ± 0.052	0.170 ± 0.020
"PAVARINI-IV"	0.539 ± 0.002	85.726 ± 0.484	19.555 ± 0.055
"PAVARINI-V"	0.125 ± 0.087	4.441 ± 0.355	0.467 ± 0.005
"PAVARINI-VI"	0.126 ± 0.021	0.452 ± 0.007	0.161 ± 0.008
"PAVARINI-VII"	0.268 ± 0.151	2.420 ± 0.028	1.568 ± 0.009
"PAVARINI-VIII"	1.619 ± 0.054	35.025 ± 0.242	57.471 ± 0.127
"PAVARINI-IX"	0.364 ± 0.143	46.038 ± 0.099	8.099 ± 0.016
"PAVARINI-X"	0.121 ± 0.004	0.419 ± 0.005	0.174 ± 0.004

Table S3. ^{1}H-, ^{13}C- and ^{1}H-^{13}C HMBC data for two QMTs (maytenin and pristimerin) identified in root barks from *Maytenus ilicifolia* (600 MHz, $CDCl_3$).

Biosynthesis	Maytenin (1)		Pristimerin (2)	
	^{1}H/^{13}C δ ppm	HMBC	^{1}H/^{13}C δ ppm	HMBC
1	6.47 (*d*, *J* 0.8 Hz, 1H, H-1); 119	-	6.48 (*d*, *J* 0.8 Hz, 1H); 119	-
2	178.4	-	178.4	-
3-OH	145.0	H1, H23	145.0	H1, H23
4	117.1	H6, H23	117.1	H6, H23
5	127.7	H1, H7, H23	127.7	H1, H7, H23
6	6.95 (*dd*, *J* 7.2 and 0.8 Hz, 1H); 134	-	6.98 (*dd*, *J* 7.1 and 0.8 Hz, 1H); 134.2	-
7	6.28 (*d*, *J* 7.2 Hz, 1H); 118	-	6.31 (*d*, *J* 7.1 Hz, 1H); 118	-
8	170.9	H1, H23	170.9	H1, H23
9	42.1	H6, H23	42.1	H6, H23
10	164.6	H1, H7, H23	164.6	H1, H7, H23
12	29.9	-	29.4	H27
13	40	-	39.0	H27
18	43.3	-	44.5	H27
22a	1.79 (*d*, *J* 14.5 Hz, 1H); 52.5	-	-	-
22b	2.84 (brd, *J* 14.5 Hz, 1H); 52.5	-	-	-
23	2,14 (*s*, 3H); 10.1	-	2,16 (*s*, 3H); 21.3	-
25	1.38 (*s*, 3H); 38	H1	1.38 (*s*, 3H); 38.2	H1
26	1.28 (*s*, 3H); 21.5	-	1.18 (*s*, 3H); 21.4	-
27	0.92 (*s*, 3H); 19.7	-	0.47 (*s*, 3H) 18.4	-
28	0.94 (*s*, 3H); 32.5	-	1.03 (*s*, 3H); 32.0	-
29-CH_3O	-	-	3.49 (*s*, 3H); 51.7	179.1
30	0.95 (*s*, 3H); 15.0	-	1.11 (*s*, 3H); 32.5	-

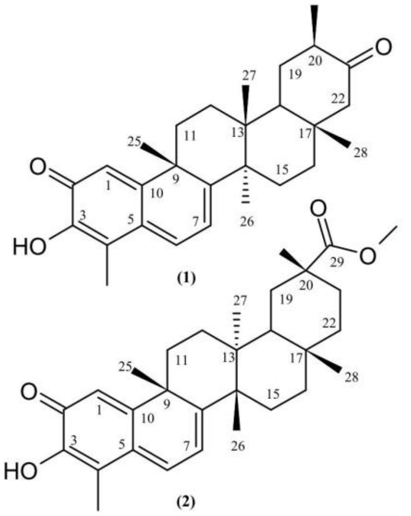

(1)

(2)

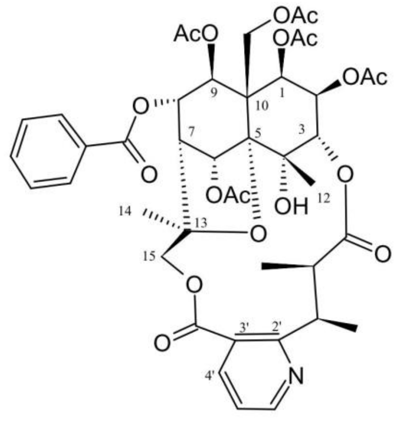

(3)

Table S4. ^{1}H-, ^{13}C- and ^{1}H-^{13}C HMBC data of sesquiterpene pyridine alkaloid (aquifoliunin E1) identified in root barks from *Maytenus ilicifolia* (600 MHz,$CDCl_3$).

**Biosynthesis**	**Aquifoliunin E-1 (3)**	
	**$^{1}H/^{13}C$ (ppm)**	**HMBC**
1	5.58 (*d*, *J* 3.5 Hz, 1H); 72.6	-
2	5.21 (*dd*, *J* 3.5 and 3.0 Hz, 1H); 68.8	-
3	4.68 (*d*, *J* 3.0 Hz, 1H); 75.6	-
4-OH	-	-
6	6.67 (*s*, 1H); 74.8	-
7	2.61 (*d*, *J* 3.5 Hz, 1H); 44.7	-
30	-	-
3′	125	H5′
4'	7.96 (*dd*, *J* 7.8; 1.8, 1H); 137	-
5'	7.18 (*dd*, *J* 7.8, 4.7 Hz, 1H); 121	-
6'	8.62 (*dd*, *J* 4.7, 1.8, 1H); 151	H4′
7'	4.6 (*q*, *J* 7.0 Hz, 1H); 36.7	-
8'	-	-
9'	-	-
10'	-	-
1-CH_3CO	-	-
2-CH_3CO	-	-
6-CH_3CO	2.15 (s, 3H)	-
8-CH_3CO	-	-
9-CH_3CO	1.83 (*s*, 3H); 19.8	-
11-CH_3CO	2.28 (*s*, 3H); 20.7	-
COPh	165	H_{orto}
ipso	129	-
ortho	7.87 (*dd J* 8.2 and 1.1 Hz, 2H); 129.2	H_{meta}
meta	7.38 (*dd*, *J* 7.4 and 8.2 Hz, 2H); 128	H_{para}, H_{ortho}
para	7.52 (*t*, *J* 7.4 Hz, 1H); 133	H_{meta}

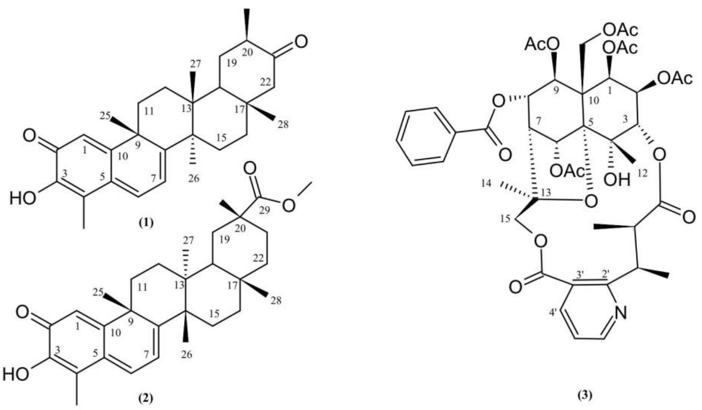

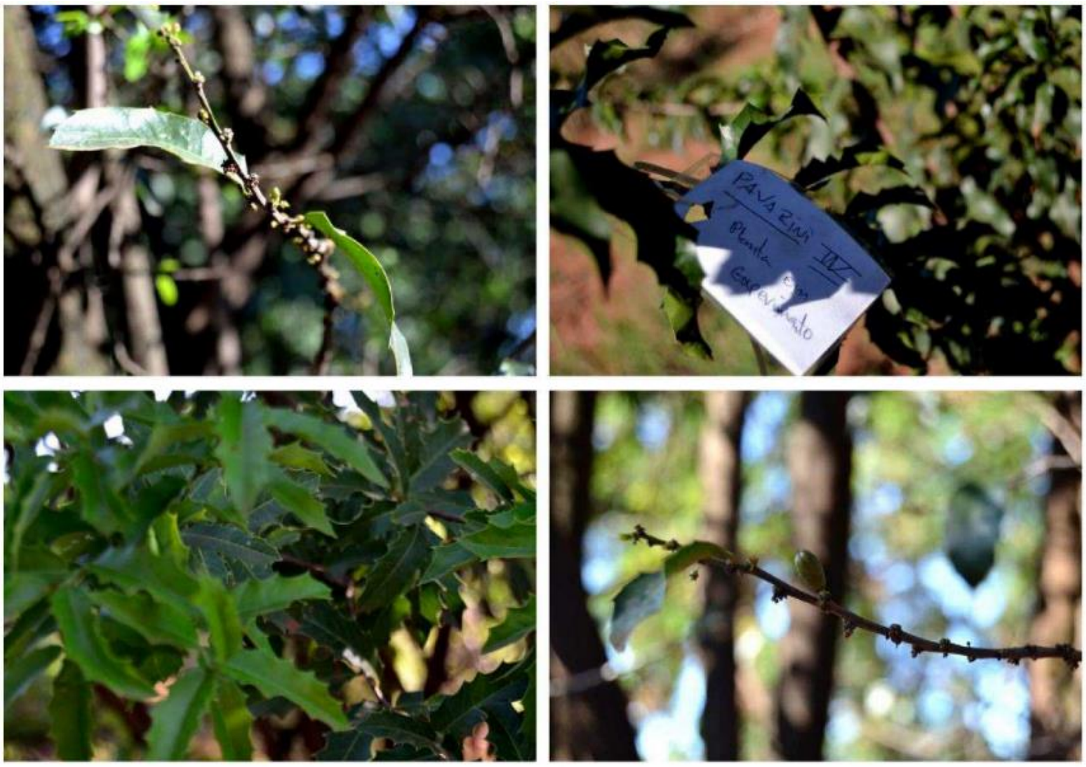

Figure S2. Photographs of *Maytenus ilicifolia* individuals and clones from the *ex situ* collection of GH-SPS. Top left displays reproductive structures. Top right is a close of the coding. Bottom left captures the vegetative aerial parts. In bottom right, a fruit is in the center.
